# Identification of Serum Monocyte Chemoattractant Protein-1 and Prolactin as Potential Tumor Markers in Hepatocellular Carcinoma

**DOI:** 10.1371/journal.pone.0068904

**Published:** 2013-07-18

**Authors:** Who-Whong Wang, Soo Fan Ang, Rajneesh Kumar, Charmain Heah, Andi Utama, Navessa Padma Tania, Huihua Li, Sze Huey Tan, Desmond Poo, Su Pin Choo, Wan Cheng Chow, Chee Kiat Tan, Han Chong Toh

**Affiliations:** 1 Department of Medical Oncology, National Cancer Centre Singapore, Singapore, Singapore; 2 Department of Gastroenterology & Hepatology, Singapore General Hospital, Singapore, Singapore; 3 Duke-NUS Graduate Medical School, Singapore, Singapore; 4 Mochtar Riady Institute for Nanotechnology, Jakarta, Indonesia; 5 Department of Clinical Research, Singapore General Hospital, Singapore, Singapore; 6 Department of Clinical Trials and Epidemiological Sciences, National Cancer Centre Singapore, Singapore, Singapore; The University of Hong Kong, Hong Kong

## Abstract

Early diagnosis of hepatocellullar carcinoma (HCC) remains a challenge. The current practice of serum alpha-fetoprotein (AFP) measurement is inadequate. Here we utilized a proteomic approach to identify novel serum biomarkers for distinguishing HCC patients from non-cancer controls. We profiled the serum proteins in a group of 58 resectable HCC patients and 11 non-HCC chronic hepatitis B (HBV) carrier samples from the Singapore General Hospital (SGH) using the RayBio® L-Series 507 Antibody Array and found 113 serum markers that were significantly modulated between HCC and control groups. Selected potential biomarkers from this list were quantified using a multiplex sandwich enzyme-linked immunosorbent assay (ELISA) array in an expanded SGH cohort (126 resectable HCC patients and 115 non-HCC chronic HBV carriers (NC group)), confirming that serum prolactin and monocyte chemoattractant protein-1 (MCP-1) were significantly upregulated in HCC patients. This finding of serum MCP-1 elevation in HCC patients was validated in a separate cohort of serum samples from the Mochtar Riady Institute for Nanotechnology, Indonesia (98 resectable HCC, 101 chronic hepatitis B patients and 100 asymptomatic HBV/HCV carriers) by sandwich ELISA. MCP-1 and prolactin levels were found to correlate with AFP, while MCP-1 also correlated with disease stage. Subsequent receiver operating characteristic (ROC) analysis of AFP, prolactin and MCP-1 in the SGH cohort and comparing their area under the ROC curve (AUC) indicated that neither prolactin nor MCP-1 on their own performed better than AFP. However, the combination of AFP+MCP-1 (AUC, 0.974) had significantly superior discriminative ability than AFP alone (AUC, 0.942; *p*<0.001). In conclusion, prolactin and MCP-1 are overexpressed in HCC and are conveniently quantifiable in patients’ sera by ELISA. MCP-1 appears to be a promising complementary biomarker for HCC diagnosis and this MCP-1+AFP model should be further evaluated as potential biomarker on a larger scale in patients at-risk of HCC.

## Introduction

Hepatocellullar carcinoma (HCC) is the sixth most common cancer worldwide (fifth most common cancer in men and the seventh in women) and the third leading cause of cancer deaths worldwide, with East and South-East Asia carrying the largest HCC burden [1], [2]. Its increased incidence is attributable to an increased prevalence of hepatitis B and C virus infections [2]. The number of new cases per year worldwide is estimated to be 748,000 (10.8 per 100,000) [1]. The outcome of patients with HCC is dismal and majority of patients present in the late stages when therapy has modest benefit. Only about 30% of newly diagnosed HCCs are detected in the early stages when potentially curative treatment such as surgical resection or liver transplantation is feasible and 5-year survival rates range from 50–70% [3].

Patients with liver cirrhosis are at risk of developing HCC [4]. Chronic hepatitis B (HBV) and C (HCV) infections are associated with about 70% and 20% of HCC in the Asian and African populations respectively [2], [4]. This provides an opportunity for close surveillance and early diagnosis in an at-risk population for which early detection may permit interventions that reduce cancer-specific mortality [5]. The American Association for the Study of Liver Disease guidelines recommended the use of periodic examination of serum alpha-fetoprotein (AFP) levels and abdominal ultrasound scans as the main screening tools for early detection of HCC in chronic liver disease patients, but the accuracy and usefulness of these tests remain questionable [6]. At a commonly adopted cut-off value of 20 ng/ml, AFP has a sensitivity ranging from 49 to 71% and specificity from 49 to 86% in HCCs smaller than 5 cm [7]–[9]. Furthermore, it was also found to exhibit no prognostication value in a group of patients with cirrhosis with a single HCC of ≤3 cm in diameter [10]. Ultrasound scanning, on the other hand, has a sensitivity of 65 to 80% and a specificity of 90% when used as a screening tool. However, its accuracy in nodular cirrhotic livers is not well defined [11]. Hence, there is still a need to identify biomarkers that can replace or complement AFP and ultrasound scanning for early diagnosis of HCC.

To be clinically useful, a diagnostic biomarker should be easily measured non-invasively in easily assessable body fluids such as serum and urine. In HCC, serum AFP-L3 and Des-gamma-carboxy prothrombin (DCP) are two of the most studied alternative HCC tumor markers, and may be more effective than AFP alone in differentiating HCC from non-malignant hepatopathy and predicting prognosis [12], [13]. However, their superiority in detecting early stage HCCs remain doubtful [14], [15]. Transforming growth factor-beta1 (TGF-β1) and serum vimentin have also been proposed as potential biomarkers for small-sized HCC tumors [16], [17]. Serum SELDI-TOF proteomic signature, alone or in combination with AFP marker, may also be a promising tool for HCC screening in an at-risk population with liver cirrhosis due to its high sensitivity and specificity [18]. In separate reports, serum glypican-3 and human telomerase reverse transcriptase (hTERT) mRNA were also found to be increased in patients with HCC [19], [20]. While Llovet JM *et al* demonstrated that a three-gene set comprising glypican-3, LYVE1 (lymphatic vessel endothelial hyaluronan receptor-1) and survivin was able to differentially diagnose HCC from dysplastic nodule tissue with high accuracy [21]. Recently, efforts by Jain *et al* showed methylation of the 5′-end of the glutathione S-transferase π 1 (GSTP1) gene promoter in tissues as a potential HCC marker to identify HCC among the at-risk hepatitis and cirrhosis patients [22]. Most recently, strong evidence had been presented to show that serum Dickkopf-1 (DKK1) could be used as a complementary biomarker for AFP for significantly superior diagnosis capability in detecting early HCC than AFP alone [23]. However, more studies are needed to validate these candidate HCC biomarkers and confirm their predictive and/or prognostic values. We therefore participated in the effort to identify novel HCC biomarkers that will improve the diagnosis of early HCC over the current screening practice of serum AFP measurements.

Enzyme-linked immunosorbent assay (ELISA)-based methods are considered to be amongst the most robust platforms for biomarker discovery and are known for their high degree of sensitivity [24]. Recent advancement in protein array technology has created a high-throughput platform for biomarker screening by ELISA. In this study, we employed the Raybiotech L-Series 507 antibody array platform, a novel antibody array that simultaneously detects 507 serum proteins, to identify potential predictive markers for HCC [25]. Here, we report the identification of two novel serum biomarkers, namely prolactin and monocyte chemoattractant protein-1 (MCP-1) that were significantly elevated in patients with resectable HCC compared to non-HCC chronic hepatitis B (HBV) carriers. We also demonstrate that one of these markers, MCP-1, may be complementary to AFP to improve the diagnosis of HCC in at-risk patients.

## Materials and Methods

### Ethics Statement

All procedures for informed consent, data collection and privacy protection were approved by the SingHealth Centralised Institutional Review Board for the Singapore General Hospital (SGH) cohort (approval number 2009/932/B for utilizing archived HCC patient serum samples obtained from the SingHealth Tissue Repository and number 2010/510/B for serum collection from non-HCC HBV carriers) and by The Committee on Health Research Ethics for the Mochtar Riady Institute for Nanotechnology (MRIN) cohort (approval number 003/MI/EC/2007). All adult patients gave written informed consent prior to serum collection. For the single HCC patient who was under 18 years of age at the time of serum collection in the SGH cohort, written consent was obtained from the legal guardian on behalf of the patient.

### Patients

From 2000 to 2011, serum from 126 patients with completely resected HCC and 115 non-HCC chronic HBV carriers (NC group) were collected from the Department of General Surgery and the Department of Gastroenterology and Hepatology, SGH respectively. All 126 HCC patients underwent hepatectomy in SGH. The histology of the resected specimens confirmed the diagnosis of HCC, and the size of tumors, presence or absence of cirrhosis in non-cancerous tissues, were routinely reported by pathologists. Table 1 summarizes the patient and tumor characteristics. These HCC patients had either pathological stage I/II HCC (S1 group, n = 93) or pathological stage III/IV HCC (S3 group, n = 33) according to AJCC Cancer Staging Manual 6^th^ edition published by the American Joint Committee on Cancer (AJCC) [26]. The patients’ ages ranged from 15–86 years with a median age of 64. Sixty-seven patients (53.2%) had HBV infection, 9 (7.1%) had hepatitis C (HCV) infection, 1 (0.8%) had both HBV and HCV infection, the remaining 49 (38.9%) were hepatitis-negative. One hundred and twenty-four patients had Child-Pugh class A liver function (98.4%) and the remaining 2 had Child-Pugh class B liver function. Most patients had Eastern Cooperative Oncology Group (ECOG) performance status of 0–1 (91%) while 5 patients (9%) had ECOG performance status of 2. Fifty-six patients (44.4%) demonstrated histological evidence of liver cirrhosis. Thirty-six patients (28.6%) had tumor size less than 3 cm while 23 patients (18.3%) had tumor size of 10 cm or more. The rest (53.1%) had tumor size of between 3 and 10 cm. In addition, each patient’s Barcelona Clinic Liver Cancer (BCLC) stage was calculated [27]. Most patients had BCLC stage A (n = 82, 65.1%), 19 had BCLC stage B (15.1%), 24 had BCLC C (19%) and 1 had BCLC D (0.8%). For the corresponding non-HCC control group (NC group), all 115 patients had chronic HBV infection. The patients’ ages ranged from 21–73 years with a median age of 52. These patients represented the at-risk population who were monitored by routine follow-up visits. All of these patients had ECOG scores of 0, normal AFP levels (<20 ng/ml) and Child-Pugh class A liver function. A follow up visit six months after serum collection confirmed these HBV carriers’ non-HCC status.

**Table 1:** Demographics of patients (SGH cohort).

Characteristic	HCC (n = 126)		NC (n = 115)	
	n	Frequency	n	Frequency
Gender				
Female	30	23.8%	40	36.4%
Male	96	76.2%	70	63.6%
Hepatitis Infection				
Hepatitis B	67	53.2%	115	100.0%
Hepatitis C	9	7.1%	0	0.0%
Hepatitis B+C	1	0.8%	0	0.0%
Non-Hepatitis	49	38.9%	0	0.0%
AFP				
<20 ng/ml	57	47.5%	110	100.0%
≥20 ng/ml	63	52.5%	0	–
Cirrhosis				
No	70	55.6%	–	–
Yes	56	44.4%	–	–
Child-Pugh Score				
A	124	98.4%	115	100%
B	2	1.6%	–	–
AJCC Stage				
I	70	55.6%	–	–
II	23	18.3%	–	–
III	32	25.4%	–	–
IV	1	0.8%	–	–
BCLC Score				
A	82	65.1%	–	–
B	19	15.1%	–	–
C	24	19%	–	–
D	1	0.8%	–	–
Tumour Size				
Size ≤3 cm	36	28.6%	–	–
3 cm<Size <10 cm	67	53.2%	–	–
Size ≥10 cm	23	18.3%	–	–

Serum samples from a separate, independent cohort of Indonesian patients at various disease stages from Mochtar Riady Institute for Nanotechnology (MRIN), Indonesia, were used for further validation of serum MCP-1 levels. This cohort consisted of: (1) HCC group (n = 98), (2) chronic viral hepatitis patients with evidence of transaminitis (CH group, n = 101), and (3) asymptomatic hepatitis carriers (AC group, n = 100: 65 HBV +35 HCV carriers).

### Serum Protein Profiling

Soluble proteins in a subset of 58 HCC (39 from S1 group, 19 from S3 group) and 11 non-HCC control serum samples from the SGH cohort were profiled using the RayBio® L-Series 507 Biotin Label-based Antibody Array system (RayBiotech, GA, USA). Briefly, proteins in the serum samples were biotinylated, followed by spin filtering to remove excess biotin. The biotinylated samples were dialyzed with PBS and added to the array membranes. Biotinylated proteins captured by the membrane-bound antibodies were detected by incubation with HRP-streptavidin and analysis by a chemiluminescence imaging system. Normalization was performed using the signal of internal controls on each protein array chip. Quality control was performed with the removal of proteins detected below the raw signal intensity of 50, which was twice the maximum intensity of the negative control probes. Significance testing was performed by *t*-Test and fold-change was cut-off at 2.0.

### Serum Protein Quantification

To validate data from the L Series 507 array analysis, selected proteins from the list of significantly modulated serum protein markers detected in the array analysis were quantified using multiplex sandwich ELISA arrays (Custom Quantibody Array, Raybiotech, GA, USA) according to the manufacturer’s instructions in the entire SGH cohort of 126 HCC patients (the 58 samples used in profiling array plus additional 68 samples) and in 115 non-HCC hepatitis B carriers (the 11 NC group samples used in the profiling array plus additional 104 samples). All samples were run in quadruplicates.

Serum MCP-1 levels in the MRIN cohort were analyzed in duplicates by sandwich ELISA using a matched antibody pair and recombinant human MCP-1 according to the manufacturer’s instructions (RayBiotech, GA, USA). Optical density was measured using Microplate Imaging System (BioRad, CA, USA). A four-parameter logistic (4-PL) curve-fit consisting of seven standard concentrations was generated using Microplate Manager 5.2.1 software (BioRad). The lower limit of detection (LOD) for each marker in the assays was determined based on the average raw data of two sets of standard curves and from the average of two negative controls and their standard deviation (by calculating Average +2x Standard Deviation). Signal strengths below the LOD for each biomarker in each assay were considered as undetectable.

In the SGH cohort, serum AFP levels in the HCC patients were measured by the SGH clinical laboratory prior to the patients undergoing hepatectomy. AFP levels in the non-HCC hepatitis B carriers were measured likewise as part of the blood tests performed during routine follow-up visits.

### Statistical Analysis

The serum biomarker levels in cancer patients and controls were compared using Mann-Whitney *U* test. Correlation between serum markers was assessed using Spearman’s rank correlation coefficient (rho). The association between each serum marker and various patient demographics and clinical parameters was assessed using the Mann-Whitney *U* test. When more than two groups were involved, the Kruskal-Wallis test was used.

Univariable logistic regression was carried out to evaluate the effect of different serum makers on the development of HCC. Recurrence-free survival (RFS) was defined as the time from date of surgery to date of first relapse, or to the last follow up date for censored cases, while overall survival (OS) was defined as the time from date of diagnosis to date of death, or to the last follow up date for censored cases. Kaplan-Meier survival curves were used to estimate RFS and OS and log-rank test was used to assess if the survival function between groups were different. The 95^th^ percentile of the non-HCC group values was rounded to the nearest one decimal point and adopted as the upper limit of normal (ULN) for each biomarker. The biomarkers were dichotomized at their ULN values and survival functions compared. Univariable Cox regression was carried out first to evaluate the effect of individual factor on RFS and OS. Subsequently, reduced model selection was carried out to select a multivariable model using a backward stepdown by applying stopping rule of the Akaike’s information criterion (AIC). Proportional hazards assumptions were verified systematically for all proposed models.

The area under the Receiver Operating Characteristic (ROC) Curve was reported to evaluate the ability of the potential serum markers in discriminating HCC patients from the controls. Multivariable logistic regression was performed to select the most suitable diagnostic model using a combination of serum markers. Clustering of these markers based on Spearman correlation was performed to reduce the number of variables to be included in the final model. Calibration plot was generated to explore the performance characteristics of this model by means of bootstrapping of 200 samples. Likelihood ratio testing of nested models was performed to compare our final diagnostic model to the other models including a subset of serum markers. An adequacy index was used to quantify the percentage of the variation explained by a subset of these predictors compared with the information contained in the full set of predictors in our final diagnostic model by means of log-likelihood.

All analyses were done using R 2.14.0 (http://www.R-project.org) and STATA 11 (STATA Corporation, College Station, TX, USA), and all tests were two-sided with a significance level of 0.05.

## Results

### Identification of Potential HCC Serum Markers

Using RayBio® L-Series 507 Antibody Array that simultaneously detects the levels of 507 proteins, we profiled the serum proteins in a subset of 58 HCC patients (39 from S1 group, 19 from S3 group) and 11 non-HCC HBV carriers from the SGH cohort to detect potential biomarkers for early detection of HCC. By comparing the profiles of patients in the HCC groups to the NC group, we identified distinct patterns that may be specific and potentially predictive for HCC. 145 and 134 proteins were significantly modulated between HBV carriers and the S1 and S3 groups of HCC patients respectively. Among these markers, we found that 113 proteins were significantly and consistently up- or down-modulated in both S1 and S3 groups relative to the NC group. Several of these proteins are known to be associated with chemotactic activities, immunomodulation, angiogenesis, cell adhesion and tumor suppression (Table S1).

### Marker Selection and Validation

**Figure 1:**
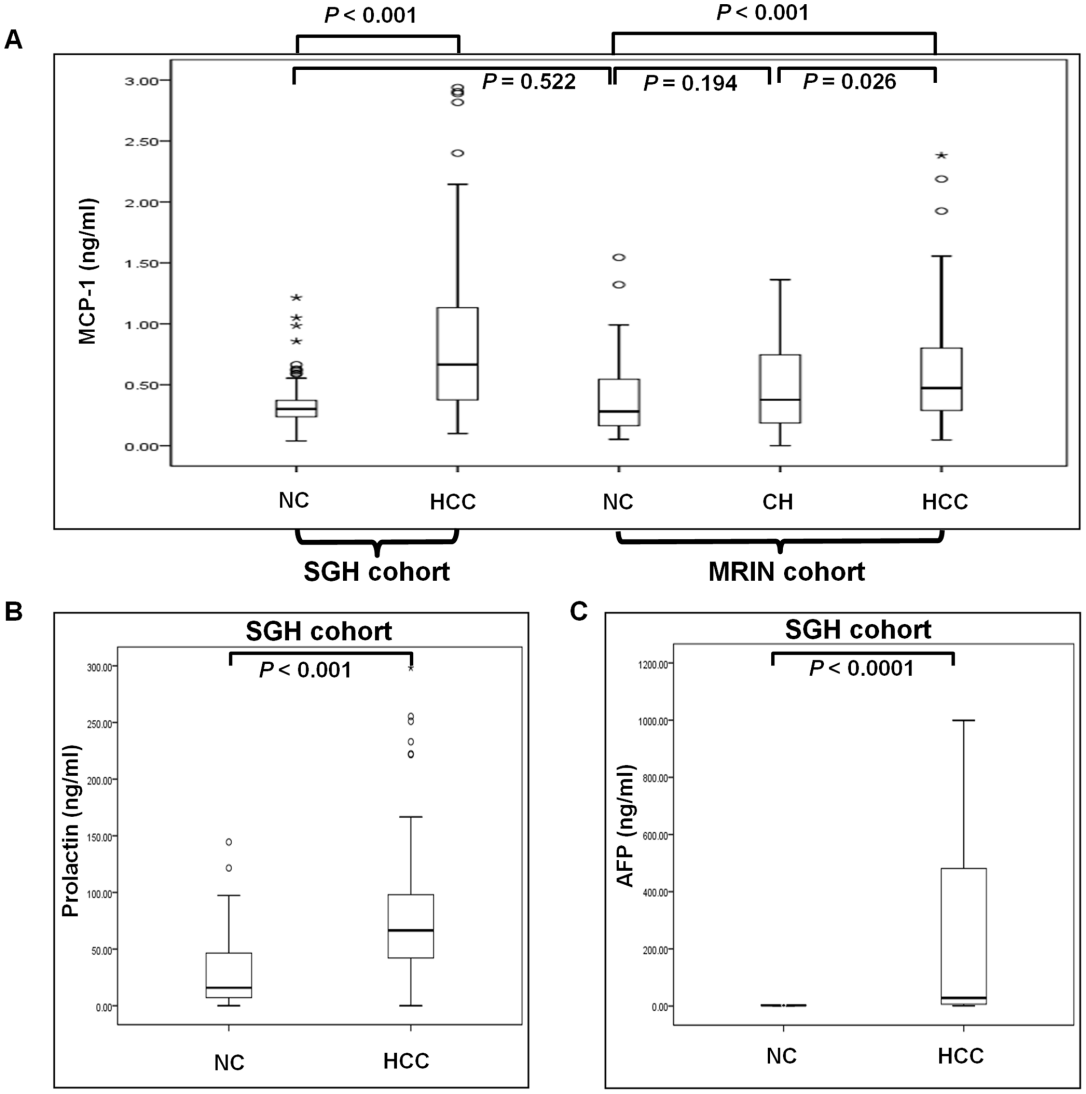
Comparison of serum MCP-1, prolactin and AFP levels in HCC and non-HCC patients. Serum concentrations of (A) MCP-1, (B) prolactin and (C) AFP in non-HCC chronic hepatitis B carriers (NC group, n = 115) and HCC patients (HCC group, n = 126) in the SGH cohort of patients were analyzed by multiplex sandwich ELISA (Quantibody Array). Serum MCP-1 concentrations in asymptomatic HBV/HCV carriers (AC group, n = 100), chronic hepatitis patients with evidence of transaminitis (CH group, n = 101) and HCC patients (HCC group, n = 98) in the MRIN cohort (A) were analyzed by sandwich ELISA. The boxes represent the central 50% of the data, spanning between the 25^th^ and 75^th^ percentiles and the horizontal line within each box indicates the median. The cut-off points were: 1.5×IQR above 75^th^ percentile (upper limit) and 1.5×IQR below 25^th^ percentile (lower limit). Values beyond the cut-off points were considered as outliers and are represented by the dots. Comparison of biomarker values between groups was performed using the Mann-Whitney *U* test.

We selected 5 proteins from the list of 113 potential markers for further validation by ELISA quantification in the SGH cohort using the Custom Quantibody Array, namely MCP-1, prolactin, angiostatin, interferon gamma inducible T cell alpha chemoattractant (I-TAC) and tissue inhibitor of metalloproteinases-4 (TIMP-4) that showed high fold changes and for which quantification by the multiplex sandwich ELISA array was possible. Consistent with the antibody array results, Mann-Whitney *U* test showed that serum samples from HCC patients (n = 126) had significantly higher levels of MCP-1 (median = 0.67 ng/ml; interquartile range, IQR, 0.37–1.14 ng/ml) and prolactin (median = 66.54 ng/ml; IQR, 41.79–98.32 ng/ml) compared to the NC group (n = 115) that had median values of 0.30 ng/ml (IQR, 0.24–0.37 ng/ml; p<0.001) and 15.85 ng/ml (IQR, 7.0–46.58 ng/ml; p<0.001) respectively (Figure 1). Concentration differences of the remaining three markers were insignificant and thus were excluded from subsequent analysis (not shown).

To test the consistency of our observation that MCP-1 was significantly elevated in HCC patients, we evaluated serum MCP-1 levels in an independent cohort of Indonesian patient samples consisting of sera obtained from 98 HCC patients, 101 chronic hepatitis B patients with evidence of transaminitis (CH group) and 100 non-HCC HBV/HCV carriers (AC grouop). We performed conventional sandwich ELISA to quantify serum MCP-1 levels and observed significantly higher MCP-1 concentration in the HCC patients (median = 0.47 ng/ml; IQR, 0.29–0.80 ng/ml) relative to CH (median = 0.38 ng/ml; IQR, 0.17–0.75 ng/ml; p = 0.026) and AC (median = 0.28 ng/ml; IQR, 0.16–0.55 ng/ml; p<0.001) groups (Figure 1A). We also found that the difference in median MCP-1 concentrations between the AC and CH groups was statistically insignificant (increment of 0.1 ng/ml; p = 0.194). Since the ELISA method used for assaying the MRIN cohort was different from the method adopted for the SGH cohort, although with the same underlying principle, we tested the sensitivity and consistency between both methods by performing Mann-Whitney *U* test to compare the MCP-1 levels detected in the non-HCC hepatitis carrier reference groups of both cohorts. The resulting p-value of 0.552 showed that the sensitivity of both ELISA methods were comparable (Figure 1A).

In the SGH cohort, serum AFP level data was available for 110 of the NC group and 120 of the HCC patients. AFP levels were <20 ng/ml in all of these non-HCC hepatitis B carriers. In the HCC patients group, AFP level was elevated above 20 ng/ml in 52.5% of patients (63/120 patients, Table 1). The serum AFP levels in HCC patients had a median value of 27 ng/ml (IQR, 1.8–31815 ng/ml) which was significantly higher than in the NC group (median = 2.5 ng/ml; IQR, 1.1–4.8 ng/ml; p = 0.0001; Figure 1C).

### Correlation of Serum MCP-1 and Prolactin Levels with AFP

Since serum AFP, MCP-1 and prolactin were significantly elevated in HCC patients, we evaluated possible correlation among these serum markers. We performed Spearman’s rank correlation coefficient (rho) calculations on the entire cohort of SGH samples collected (n = 241) and found significant positive correlation between MCP-1 and AFP levels (rho = 0.7926, p = 0.024) and a weaker correlation between prolactin and AFP levels (rho = 0.6419, p = 0.043). Interestingly, a weak inverse correlation was found between MCP-1 and prolactin (rho = −0.269, p = 0.002; Table 2).

**Table 2:** Correlation between serum markers estimated by Spearman’s rank correlation coefficient, rho (n = 241).

	MCP-1	Prolactin	AFP	AST	ALT
MCP-1		−0.2689	0.0244	0.0699	0.0809
Prolactin	0.0024		0.0431	0.0324	−0.0621
AFP	0.7926	0.6419		0.1838	−0.0319
AST	0.4387	0.7201	0.0444		0.6498
ALT	0.3697	0.4918	0.7291	<0.0001	

* The upper part are the Spearman’s rank correlation coefficients, rho; the lower part are the p-values of the Spearman’s rank correlation test.

### Association of Serum MCP-1 and Prolactin Levels with Various Patient Demographics and Disease Characteristics

We also analyzed for effects of various clinical parameters on serum MCP-1 and prolactin levels in the SGH cohort. We found that serum levels of MCP-1 was significantly higher in patients with late stages and higher BCLC scores (p = 0.0005 and p<0.0001 respectively). Serum prolactin level, however, was not affected by these parameters (p = 0.2869 and p = 0.2145 respectively). Other clinicopathologic parameters, such as gender, viral hepatitis status and liver cirrhosis did not affect the serum levels of these two markers. We also evaluated the impact of hepatitis using serum aspartate transaminase (AST) and alanine transaminase (ALT) as indices for injury to hepatocytes. We found no association between MCP-1 and prolactin levels with liver function status (Table S2).

### Association of Clinicopathological Parameters and Serum Markers with RFS and OS

Median follow-up for the SGH cohort of patients with resected HCC was 2.3 years (IQR, 1.0–4.1 years). Median OS for these 126 patients was 5.4 years, while the median RFS was 2.4 years (Figure S1). By means of univariable Cox regression analysis, clinicopathological parameters that shown statistical significant association with RFS were ALP and PT (p = 0.028 and 0.029 respectively; Table S3). Multivariable analyses continued to show that RFS was significantly affected by both ALP and PT (p = 0.038 and 0.031 respectively). Factors associating with the OS were found to be ALP (p = 0.001), PT (p = 0.012), AJCC staging (p = 0.003) and Childs Pugh scores (p<0.001) by means of univariable anlaysis (Table S4). However, multivariable analyses showed that only ALP and PT affected the OS significantly (p = 0.001 and 0.012 respectively). We found no effect of MCP-1, prolactin and AFP levels on patient survival. The upper limit of normal (ULN) values for serum MCP-1 and prolactin were determined to be 0.62 ng/ml and 83.63 ng/ml respectively. When these ULN serum levels were used as cut-off values for analysis, we found that measurement of serum MCP-1 and prolactin levels did not predict RFS (p = 0.7318 and 0.6290 respectively) or OS outcome (p = 0.3348 and 0.2336 respectively).

### Comparison of Diagnostic Sensitivity and Specificity among MCP-1, Prolactin and AFP

**Figure 2:**
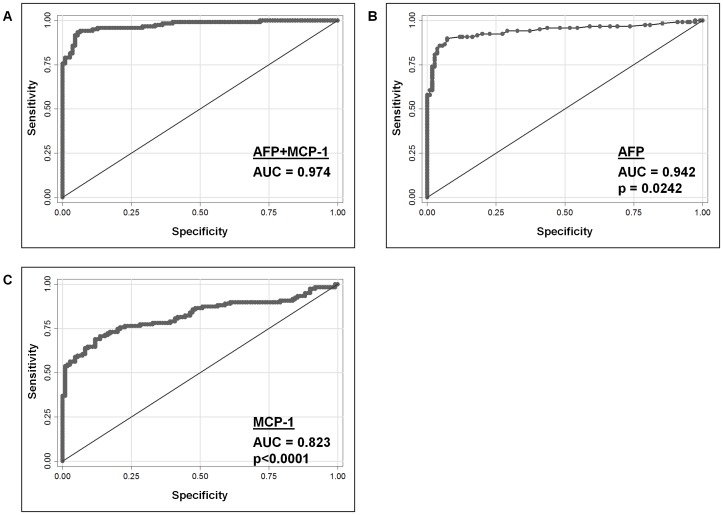
Receiver Operating Characteristic (ROC) curves. **ROC curves of different models for the SGH cohort were generated, based on samples whose MCP-1, prolactin and AFP values were available (120 HCC and 110 non-HCC controls).** Upon comparison, the AFP+MCP-1 combination (A; AUC = 0.9735) showed significantly improved sensitivity and specificity compared to AFP alone (B; AUC = 0.9415, p = 0.0242) and MCP-1 alone (C; AUC = 0.8225, p<0.0001).

To evaluate the diagnostic values of serum MCP-1 and prolactin in distinguishing HCC patients from non-HCC hepatitis B carriers in comparison with serum AFP, we performed the ROC curve analysis to determine the sensitivity and specificity of these three markers in the SGH cohort. The area under the ROC curve (AUC) for MCP-1, prolactin and AFP were calculated to be 0.823 (IQR, 0.766–0.879), 0.820 (IQR, 0.766–0.874) and 0.942 (IQR, 0.908–0.975) respectively (Figure 2). The AUC of AFP was significantly higher than both MCP-1 (p = 0.0004) and prolactin (p = 0.0002; figure not shown). Univariable logistic regression analysis also showed that the higher the values of MCP-1, prolactin and AFP, the higher the possibility of the subject having HCC, while multivariable analysis of these markers further showed that all three affected the risk of having HCC simultaneously (Table S5). Based on the ROC analysis, the optimal cut-off values and the corresponding sensitivity and specificity for AFP, MCP-1 and prolactin were calculated. At a cut-off value of 0.39 ng/ml for MCP-1, a 73.1% sensitivity and 80.9% specificity was achieved. The optimal cut-off value for prolactin was reached at 39.9 ng/ml with 77.3% sensitivity and 70.9% specificity. At the commonly adopted cut-off value of 20 ng/ml for AFP, its sensitivity and specificity were 52% and 100% respectively in the SGH cohort. Surprisingly, the optimal cut-off value for AFP in this cohort was determined to be 4.0 ng/ml, with 89.9% sensitivity and 92.7% specificity.

**Figure 3:**
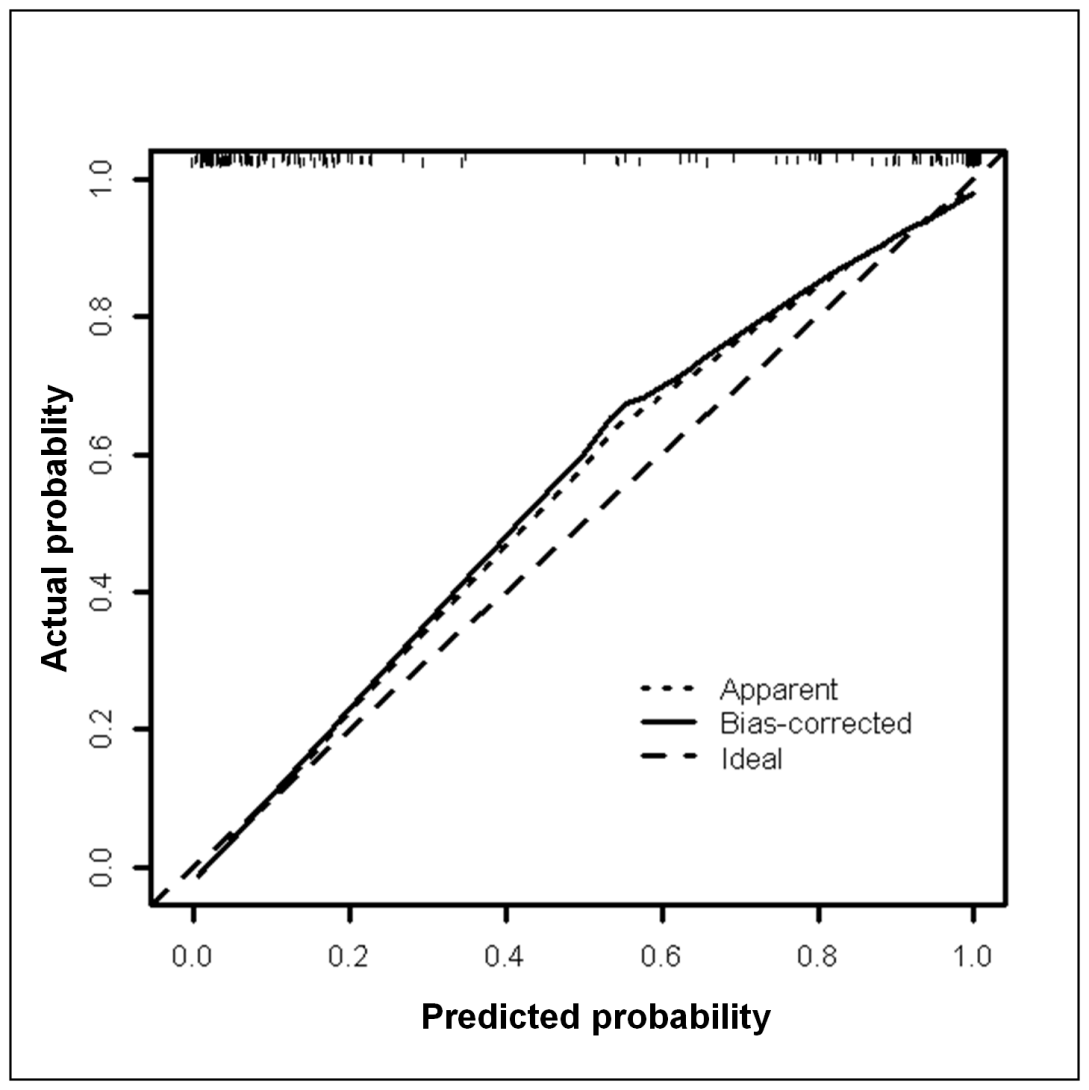
Calibration of the MCP-1+AFP diagnostic model. Calibration of the AFP+MCP-1 diagnostic model performed via bootstrapping using 200 samples. The predicted probability of HCC was found to be lower than the actual probability of HCC occurrence.

As the clustering of these factors showed a strong correlation between prolactin and AFP, the ability of discriminating HCC patients from non-HCC HBV carriers was superior when the combination of MCP-1 and AFP was used (AUC = 0.974; Figure 2A), compared to MCP-1+prolactin (AUC = 0.914; not shown). Therefore, our final diagnostic model included AFP+MCP-1 only, which was significantly superior to AFP (p = 0.0242) or MCP-1 (p<0.0001) alone for HCC detection (Figure 2). The probability of HCC detection using the AFP+MCP-1 model was calculated using the formula:
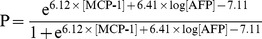



The optimum cut-off P-value was then determined. At the optimum cut-off P-value of ≥0.29 (such as at cut-off values of 4 ng/ml AFP +0.4 ng/ml MCP-1), the proposed AFP+MCP-1 model achieved a more favourable 94.1% sensitivity compared to AFP alone’s 89.9% at the same 92.7% specificity for HCC detection. Calibration of this model by means of bootstrapping using 200 samples showed that the predicted probability of HCC was lower than the actual probability of HCC occurrence (Figure 3). Compared to our final model of AFP+MCP-1, the Adequacy Index was only 78.7% for AFP (p<0.001) and 44.1% for MCP-1 alone (p<0.001, Table 3). Log likelihood ratio tests showed that the addition of MCP-1 to the AFP model improved its diagnostic value in discriminating HCC patients from non-HCC HBV carriers, and vice versa (Table 3).

**Table 3:** Comparison of the Adequacy Index and Log Likelihood ratios of diagnostic models.

Model	AUC	p-value	Likelihood	Adequacy Index
AFP+MCP-1	0.974		228.36	100.0%
AFP	0.942	<0.001	179.69	78.7%
MCP-1	0.820	<0.001	100.67	44.1%

## Discussion

HCC is a heterogeneous neoplasm with an overall poor prognosis. Currently, measurement of serum AFP and abdominal ultrasound examination are routinely used as screening tools for the early detection of HCC in chronic liver disease patients [6]. There have been many studies which suggest survival advantage and cost effectiveness of early HCC diagnosis and treatment [5], [6], [28], [29]. Yang *et al* showed that screening the high-risk population, such as patients with hepatitis B and C, with a serum AFP test and real-time ultrasound examination can detect HCC in the early stages, increasing the resection rate and prolonging the survival time [28]. Nevertheless, the accuracy and usefulness of serum AFP and ultrasound liver have their own limitations. Thus there is a real need to identify reliable biomarkers with higher degree of sensitivity and specificity for early detection of HCC.

Early biomarker discovery methods involved protein separation-based approaches, most frequently gel-based techniques such as two-dimensional polyacrylamide gel electrophoresis (2-D-PAGE). However the classical 2-D electrophoresis approach has comparably low sensitivity, especially in detecting low abundance proteins, and requires large amounts of tissue samples as well as subsequent labour-intensive procedures to optimally identify the biomarker. These techniques have evolved with the use of mass spectrometry, which allows the identification of many proteins with high sensitivity, although the sensitivity decreases with increased complexity of the samples. Mass spectrometry allows the relative comparison of proteins in different samples but the method is not quantitative. In recent years, techniques such as matrix-assisted laser desorption/ionization (MALDI) and surface-enhanced laser desorption ionization (SELDI) that later incorporated time-of-flight (TOF) mass spectrometry have achieved high resolution profiling. However, despite the significant increase in throughput and sensitivity, they do not eliminate the need for extensive subsequent procedures to definitively identify the differentially expressed protein/peptides in tumor tissues or serum [24], [30].

The ELISA-based proteomics platform is considered by some to be the most versatile “omics” method for biomarker discovery [24]. However, it is limited by the availability of well-validated antibodies [31]. This requirement also makes it ill-suited for detecting modulations of uncharacterized proteins. However, the recent advancement in protein microarrays has now given the ELISA-based approach the capacity for high-throughput and improved sensitivity. By adopting such platform to screen for potential serum markers, we identified 113 potential serum markers that were significantly modulated between HCC and control groups. Upon validation by ELISA quantification of five selected markers, we identified two novel markers, namely MCP-1 and prolactin, to be significantly elevated in sera of HCC patients who were eligible for potentially curative measures such as resection or liver transplant. The significant elevation in serum levels of these two markers in resectable HCC patients suggests their potential role as biomarkers in augmenting diagnosis and surveillance of HCC in at-risk patients. To the best of our knowledge, this is the first time that the RayBio® L-Series 507 Antibody Array has been used to identify potential cancer biomarkers, and that has been validated by multiplex sandwich ELISA arrays.

The finding of significant correlation between serum AFP and MCP-1 as well as prolactin in our SGH cohort suggested a possible synergistic mechanism of action and oncogenic pathways in HCC or in/with its surrounding microenvironment. Furthermore, our result reflected no correlation between serum concentrations of MCP-1 and prolactin with other clinicopathological features in the HCC patients, including liver cirrhosis, portal vein thrombosis, viral hepatitis status, BCLC stage, and gender. Hence it is unlikely that MCP-1 and prolactin are surrogate serum markers for these factors and more likely to be HCC-specific in the resectable HCC patients.

We next investigated the diagnostic capabilities of MCP-1 and prolactin by performing ROC analysis and comparing the resulting AUC with that of AFP in the SGH cohort. Our result showed that although all three markers were sufficiently competent (AUC >0.8), neither MCP-1 (AUC = 0.823) nor prolactin (AUC = 0.820) on their own performed better than AFP alone (AUC = 0.942). Most studies adopted a cut-off value of 20 ng/ml for AFP, which gave lower sensitivity and/or specificity especially in HCCs smaller than 5 cm [32]. In this study, our finding of 4 ng/ml as the optimum cut-off level for AFP in our local population, a value that is well within the normal range, further emphasized the inadequacy of using AFP alone for HCC diagnosis in its current form. Furthermore there is also wide overlapping between HCC and chronic liver disease. The individual marker’s limitation in the sensitivity and specificity could be overcome with the use of combinations of biomarkers. We therefore tested different combinations of these three markers and successfully identified a combination, AFP+MCP-1, that showed significantly superior diagnostic ability than AFP alone (AUC = 0.974 versus 0.942, p = 0.0242). With AFP known to have a significantly lower positive predictive value in viral-related HCC than non-viral HCC [33], it is important to establish biomarkers for better surveillance and detection of virally-related HCC in at-risk individuals, especially in China and most of Asia, where Hepatitis B is the predominant aetiology for HCC. Pending confirmation by further prospective study, we propose that the AFP+MCP-1 model may answer that need.

Following our identification of the AFP+MCP-1 model, we proceed to confirm our observation of significantly elevated serum MCP-1 level in HCC patients by performing sandwich ELISA analysis of serum MCP-1 levels in a separate and independent cohort of Indonesian patients from MRIN that included 98 patients with HCC, 101 patients with chronic hepatitis B or C, and 100 asymptomatic HBV/HCV carriers. The findings were consistent with our initial results. Due to the use of different ELISA techniques in quantifying serum MCP-1 levels in our two patient cohorts, we compared the serum MCP-1 levels in the non-HCC hepatitis virus carrier groups of both SGH and Indonesian cohorts, which represented the baseline levels that were least likely to be influenced by any clinicopathological conditions. We hypothesized that for a serum marker that was independent of clinicopathological parameters other than HCC status, MCP-1 levels in the control groups should be consistent between the two cohorts. Therefore, MCP-1 levels detected by different methods but with similar sensitivity should not be significantly different. The result showed that the levels detected by the two different ELISA methods (median levels of 0.302 ng/ml in the SGH cohort versus 0.281 ng/ml of the MRIN cohort) were comparable and robust, with a p-value of 0.552.

The value of diagnostic biomarkers would be enhanced if they also possess prognostication value. We therefore investigated the potential of adopting serum MCP-1 and prolactin as prognostic biomarkers by analysing for their associatuion with patients’ RFS and OS. Our result showed no correlation between these markers and both RFS and OS. Neither did we observe correlation between serum AFP values and both RFS and OS. This result is consistent with the findings by other groups, thus reiterating the limitation of AFP as a prognostic HCC biomarker [10], [34].

Our finding also suggests the involvement of MCP-1 and prolactin in the development of HCC. Elevated serum MCP-1 as a cancer diagnostic marker has been suggested for other malignancies, such as in pancreatic [35] and ovarian [36] cancers. However, MCP-1 may also be downregulated in certain cancers, as observed in a Japanese study in gastric cancer [37]. Inflammation caused by innate immune cells has been recognized as one of the hallmarks of cancer development [38]. Since MCP-1 is a member of the small inducible gene (SIG) family, and plays a role in the recruitment of monocytes to sites of injury and infection, its expression may plausibly be associated with HCC development. There are several studies showing that MCP-1 expression is associated with HCC progression [39]. While association between MCP-1 and liver damage has been implied due to the findings that specific non-malignant cells are major source of MCP-1, such as the hepatic stellate cells that are known to be involved in the development of fibrosis and cirrhosis [40], [41], such MCP-1 expression occurs during more profound acute liver injury [42], [43]. In an earlier study of a cohort of alcoholic liver disease patients, it was shown that serum MCP-1 level was only significantly elevated in severe alcoholic hepatitis patients relative to that of healthy controls, mild alcoholic hepatitis and inactive cirrhosis patients [44]. Only an insignificant increasing trend was observed among the healthy controls, inactive cirrhosis and mild alcoholic hepatitis patient groups. In our own cohort of resectable HCC patients, although cirrhosis was seen in 44.4% of the HCC group, majority of the HCC patients had Child-Pugh class A score (98.4%) to suggest the presence, if any, of only minimal or mild liver inflammation and thus no significant activation of the hepatic stellate cells. Even so, the MRIN data also demonstrated that serum samples of patients without HCC but with hepatitis showed only an insignificant increasing trend in serum MCP-1 levels compared to the asymptomatic hepatitis B or C virus carriers without active hepatitis (p = 0.194). On the other hand, it is also known that in HCC, HCC cells and cancer-associated fibroblasts are prominent contributors of MCP-1, regardless of whether the liver is cirrhotic or the hepatitis status [45], [46]. Therefore, the elevated serum MCP-1 levels in the HCC patients in this project were likely to be predominantly expressed by HCC tumors and the HCC-associated cells, thus showing no significant correlation with the other liver function parameters. Taken together, MCP-1 is likely involved in the HCC oncogenic process and is produced either from the tumor itself or from the surrounding stroma.

Elevation of serum prolactin, on the other hand, had also been observed in other malignancies and explored for their potential as diagnostic biomarkers, such as in lung and ovarian cancers [47]–[51]. However, although shown to be potentially useful as a member of multi-marker diagnostic panels, none of these studies could establish prolactin as an independent cancer marker. Likewise, we observed significant elevation of serum prolactin level in HCC patients but failed to establish it as an independent diagnostic HCC biomarker. Studies in the rat model had shown that prolactin promoted HCC progression while prolactin inhibition led to reduced tumor growth and longer latency [52]. Furthermore, HCC is known to highly express prolactin receptors [53]. A recent study reported by Yeh *et al* provides evidence that prolactin may promote HCC progression through the activation of the Janus kinase 2 pathway upon binding to its receptor [54].

In conclusion, we have found significantly elevated serum MCP-1 and prolactin in HCC patients and identified serum MCP-1 as a promising and potentially complementary biomarker with AFP that may offer more effective early detection of HCC. Upon further validation in larger sample size studies, measurements of their serum levels by ELISA, in particular the combination of MCP-1 and AFP, could prove to be a cheap, rapid, accessible and user-friendly method of better detection, especially in developing countries. This will potentially further lessen the huge burden of care of especially advanced HCC patients globally.

## Supporting Information

Figure S1 Kaplan-Meier survival estimates of HCC patients. Kaplan-Meier survival estimates of A. Overall survival (OS, median = 5.4 years) and B. Recurrence-free survival (RFS, median = 2.4 years) of the 126 HCC patients in the SGH cohort.
(TIF)

Table S1 List of 113 serum proteins that were found significantly modulated by the RayBio® L-Series 507 Biotin Label-based Antibody Array in a subset of HCC patients (n = 58) relative to non-cancer HBV carriers (NC, n = 11) of the SGH cohort.
(DOC)

Table S2 Association of serum MCP-1 and prolactin levels with patient demographics and disease characteristics in the HCC patients of the SGH cohort (n = 126).
(DOC)

Table S3 Summary of univariable analysis for association of factors with recurrence-free survival (RFS).
(DOC)

Table S4 Summary of univariable analysis for association with overall survival (OS).
(DOC)

Table S5 Univariable logistic regression analysis results for diagnostic ability of markers.
(DOC)
