# Supplementary material for: Identification of Serum Monocyte Chemoattractant Protein-1 and Prolactin as Potential Tumor Markers in Hepatocellular Carcinoma
Source: PLoS One. 2013 Jul 18;8(7):e68904. doi: 10.1371/journal.pone.0068904 (PMC3715515; doi:10.1371/journal.pone.0068904)
Supplement: Table S1 — List of 113 serum proteins that were found significantly modulated by the RayBio® L-Series 507 Biotin Label-based Antibody Array in a subset of HCC patients (n = 58) relative to non-cancer HBV carriers (NC, n = 11) of the SGH cohort. (DOC) [file pone.0068904.s002.doc]

Table S1. List of 113 serum proteins that were found significantly modulated by the RayBio® L-Series 507 Biotin Label-based Antibody Array in a subset of HCC patients (n = 58) relative to non-cancer HBV carriers (NC, n = 11) of the SGH cohort.

|  | Stage I vs NC (n = 11) | | | Stage III vs NC (n = 11) | | |
| --- | --- | --- | --- | --- | --- | --- |
| Serum proteins | p-value* | Fold Difference | Regulation^†^ | p-value* | Fold Difference | Regulation^†^ |
| Activin RIA / ALK-2 | 5.54E-04 | 4.384257 | up | 0.017071832 | 3.9466324 | up |
| ALCAM | 4.22E-04 | 4.025451 | up | 0.019242007 | 3.6198678 | up |
| Angiopoietin-1 | 0.012150661 | 2.1309776 | down | 0.006189216 | 2.3498816 | down |
| Angiopoietin-2 | 5.88E-05 | 29.565725 | down | 2.63E-06 | 37.235394 | down |
| Angiopoietin-like 1 | 0.006014116 | 9.166125 | down | 0.020613596 | 6.444702 | down |
| Angiopoietin-like 2 | 0.001004676 | 2.8480053 | down | 0.004739869 | 3.0806856 | down |
| Angiostatin | 2.40E-04 | 24.192965 | down | 4.02E-05 | 30.230831 | down |
| APRIL | 0.010364365 | 3.4425652 | down | 0.005632103 | 4.6581006 | down |
| beta-Catenin | 0.002674254 | 4.4219913 | down | 0.00174925 | 6.1236234 | down |
| beta-NGF | 5.54E-04 | 24.959171 | up | 4.34E-06 | 45.499466 | up |
| BMP-3 | 4.12E-04 | 4.628384 | down | 6.32E-04 | 6.359582 | down |
| BMP-3b / GDF-10 | 2.40E-04 | 7.0730076 | up | 0.001794157 | 7.7504334 | up |
| BMP-4 | 0.024733359 | 2.7510107 | down | 0.046085462 | 3.4688866 | down |
| BMP-6 | 0.006115486 | 2.623621 | down | 0.00174925 | 3.003472 | down |
| BMP-8 | 5.17E-04 | 2.634921 | down | 6.07E-04 | 3.1214225 | down |
| BMPR-IA / ALK-3 | 0.011923611 | 2.0647407 | down | 0.011436473 | 2.4368527 | down |
| BMPR-IB / ALK-6 | 5.68E-04 | 7.7896466 | down | 4.74E-04 | 8.825459 | down |
| BMPR-II | 0.023744222 | 5.2530136 | up | 3.18E-04 | 9.962704 | up |
| BTC | 0.001718334 | 5.424713 | up | 1.52E-04 | 7.9258246 | up |
| CCL14 / HCC-1 / HCC-3 | 0.003789137 | 3.1176383 | up | 0.0394291 | 3.4706337 | up |
| CCR2 | 0.007324959 | 2.2876592 | down | 0.00174925 | 2.9997544 | down |
| CCR4 | 1.58E-04 | 3.786775 | down | 0.001007292 | 4.4569254 | down |
| CCR6 | 5.54E-04 | 3.7409217 | down | 0.001082206 | 4.3058825 | down |
| CCR7 | 0.007132678 | 5.810497 | down | 0.011436473 | 8.27792 | down |
| CCR8 | 5.88E-05 | 18.070736 | down | 1.04E-05 | 30.860367 | down |
| CCR9 | 0.009293957 | 6.25603 | down | 0.003204854 | 13.733227 | down |
| CD27 / TNFRSF7 | 1.12E-04 | 2.7935305 | down | 0.003134112 | 2.437817 | down |
| Chem R23 | 0.002206852 | 6.500899 | down | 0.003763093 | 8.1776495 | down |
| Chordin-Like 2 | 3.14E-04 | 13.115384 | down | 7.45E-06 | 21.72265 | down |
| CNTF | 0.002301017 | 9.639625 | down | 0.002114036 | 14.37327 | down |
| Coagulation Factor III / Tissue Factor | 0.003491846 | 3.0870728 | up | 0.047514953 | 2.492917 | up |
| CRTH-2 | 1.77E-04 | 9.71354 | down | 9.95E-07 | 17.780085 | down |
| CTACK / CCL27 | 3.43E-04 | 22.34772 | down | 1.37E-06 | 56.04642 | down |
| CTLA-4 /CD152 | 0.001774932 | 2.5767279 | down | 0.002726238 | 3.0889003 | down |
| CXCR2 / IL-8 RB | 0.02219817 | 5.820886 | down | 0.016002305 | 8.713949 | down |
| CXCR4 (fusin) | 0.003921277 | 3.096289 | down | 0.002289749 | 4.0015407 | down |
| CXCR6 | 0.009210924 | 2.6266763 | down | 0.002796178 | 3.4336038 | down |
| Dkk-4 | 0.00159997 | 2.204756 | down | 0.003141874 | 2.4521205 | down |
| DR3 / TNFRSF25 | 0.001452212 | 2.2083051 | down | 0.011943771 | 2.142915 | down |
| EDG-1 | 0.029627228 | 2.3657992 | down | 0.002623626 | 2.9384909 | down |
| Eotaxin-3 / CCL26 | 0.04811491 | 2.7537165 | up | 0.048921145 | 2.921523 | up |
| ErbB3 | 0.024802877 | 2.0228503 | down | 0.04626915 | 2.3517985 | down |
| E-Selectin | 0.026867434 | 2.4099588 | down | 0.023521747 | 3.2103176 | down |
| FGF Basic | 0.003789137 | 4.2941055 | down | 1.43E-04 | 6.41573 | down |
| FGF-12 | 0.003471893 | 4.7223277 | up | 0.007910444 | 5.403457 | up |
| FGF-13 1B | 0.002301017 | 4.1913867 | down | 8.55E-05 | 7.8623085 | down |
| FGF-16 | 1.12E-04 | 4.6267266 | down | 0.001989909 | 4.9433265 | down |
| Follistatin | 1.12E-04 | 24.727993 | down | 3.77E-07 | 54.63156 | down |
| Follistatin-like 1 | 0.023744222 | 3.6710355 | down | 0.002206888 | 8.131599 | down |
| GASP-1 / WFIKKNRP | 0.007607468 | 5.618827 | down | 0.00174925 | 11.285146 | down |
| GDF-15 | 0.003575342 | 3.1563659 | down | 0.002877749 | 3.930579 | down |
| GITR / TNFRF18 | 0.046497863 | 2.2019053 | down | 0.01772535 | 2.9788427 | down |
| GITR Ligand / TNFSF18 | 3.14E-04 | 20.910658 | up | 3.22E-07 | 32.72338 | up |
| Glucagon | 2.26E-04 | 336.87476 | up | 1.37E-06 | 943.8644 | up |
| Glut2 | 8.90E-04 | 15.764309 | down | 0.003141874 | 20.183647 | down |
| Glut5 | 0.005534612 | 6.456616 | down | 0.006189216 | 11.422262 | down |
| Glypican 5 | 1.12E-04 | 14.003079 | down | 6.86E-05 | 23.781837 | down |
| Growth Hormone R (GHR) | 7.47E-05 | 51.47228 | up | 5.43E-08 | 90.68709 | up |
| HB-EGF | 4.40E-04 | 99.50416 | up | 1.17E-06 | 317.1891 | up |
| HCR / CRAM-A/B | 0.001810572 | 2.754293 | down | 0.004092613 | 3.1412864 | down |
| HRG-alpha | 0.001968705 | 5.075606 | up | 0.00160707 | 5.732473 | up |
| ICAM-5 | 2.06E-04 | 13.459108 | down | 8.15E-04 | 16.06109 | down |
| IGFBP-3 | 5.88E-05 | 4.2447658 | down | 1.62E-04 | 4.864326 | down |
| IGFBP-6 | 9.09E-05 | 7.8068833 | down | 4.13E-05 | 9.937257 | down |
| IL-1 F5 / FIL1delta | 0.002673093 | 3.161339 | down | 0.016930094 | 3.339649 | down |
| IL-2 R beta/CD122 | 0.014810744 | 3.2755136 | up | 0.011413869 | 3.6045747 | up |
| IL-3 | 0.010190249 | 2.7637644 | down | 0.012690818 | 3.5390575 | down |
| IL-3 R alpha | 1.66E-04 | 3.660657 | up | 0.009726431 | 3.183749 | up |
| IL-7 | 0.006014116 | 2.7515502 | up | 0.039283987 | 2.9152448 | up |
| IL-9 | 9.99E-04 | 4.9273868 | up | 0.022304697 | 4.033102 | up |
| IL-12 R beta 2 | 0.01197324 | 4.217657 | up | 0.012179313 | 4.2957087 | up |
| IL-16_ | 0.003739439 | 3.3332088 | up | 0.008000878 | 3.440069 | up |
| IL-17B R | 1.44E-04 | 4.0048714 | down | 0.001049086 | 5.274981 | down |
| IL-17C | 0.021778462 | 2.8298082 | down | 0.047341365 | 3.2936792 | down |
| IL-17F | 0.006685346 | 7.692996 | down | 0.001375102 | 18.10698 | down |
| IL-19 | 5.63E-04 | 2.9307745 | down | 3.94E-04 | 4.1686797 | down |
| IL-20 R alpha | 0.008317849 | 2.7608414 | up | 0.026338156 | 2.543306 | up |
| IL-29 | 0.006510845 | 5.873734 | down | 9.13E-04 | 9.505658 | down |
| Inhibin A_ | 0.001151892 | 4.727922 | down | 0.011413869 | 7.405247 | down |
| I-TAC / CXCL11 | 0.001999114 | 14.083898 | down | 2.68E-05 | 44.500362 | down |
| Leptin R | 0.002158305 | 28.971685 | up | 7.45E-06 | 97.90323 | up |
| LIF R alpha | 0.007810369 | 15.6140375 | down | 0.04831654 | 9.3125515 | down |
| MCP-1 | 0.001391582 | 11.1224985 | up | 3.26E-05 | 17.67394 | up |
| MCP-2 | 1.12E-04 | 21.008766 | up | 5.24E-06 | 33.837456 | up |
| MCP-4 / CCL13 | 4.90E-04 | 4.018702 | up | 0.00530792 | 4.686108 | up |
| M-CSF | 0.002125842 | 9.41051 | down | 8.34E-05 | 22.648375 | down |
| MDC | 0.024895865 | 4.1190896 | down | 1.52E-04 | 13.981965 | down |
| MIP-1b | 0.016995424 | 7.2420473 | down | 0.04107585 | 4.4878454 | down |
| MMP-15 | 2.26E-06 | 8.850185 | up | 0.002289749 | 6.665443 | up |
| MMP-16 / MT3-MMP | 3.14E-04 | 5.2963653 | up | 0.007155639 | 4.1683073 | up |
| MMP-24 / MT5-MMP | 1.12E-04 | 8.865335 | up | 0.0034858 | 7.216031 | up |
| Neuropilin-2 | 0.021778462 | 2.6644914 | up | 0.008126603 | 3.542673 | up |
| OSM | 0.022535803 | 4.826324 | down | 0.015476112 | 7.0012703 | down |
| Osteoactivin / GPNMB | 0.007206745 | 6.736415 | down | 0.015299901 | 10.521908 | down |
| PARC / CCL18 | 0.006014116 | 2.505305 | down | 0.033951897 | 2.8955617 | down |
| PD-ECGF | 0.030819992 | 2.5030413 | up | 0.04044843 | 3.0131412 | up |
| PDGF-BB | 0.002998735 | 2.129057 | up | 0.008229504 | 2.1678731 | up |
| PDGF-C | 0.002506603 | 4.126036 | up | 0.002351518 | 4.600902 | up |
| Prolactin | 0.04313515 | 3.5096693 | up | 0.009566063 | 6.61502 | up |
| S100 A8/A9 | 0.001810572 | 4.146749 | up | 0.013146365 | 4.4313707 | up |
| Smad 1 | 2.40E-04 | 9.156955 | down | 6.03E-04 | 10.801578 | down |
| Soggy-1 | 0.004300259 | 3.6736603 | up | 0.033530947 | 3.9273314 | up |
| TGF-beta RIII | 5.17E-04 | 6.717642 | up | 0.013775715 | 7.1406317 | up |
| Thrombospondin (TSP) | 2.26E-06 | 5.318565 | down | 2.95E-05 | 5.9643373 | down |
| TIMP-4 | 1.77E-04 | 20.829704 | up | 4.99E-06 | 38.853188 | up |
| TRADD | 0.00159997 | 7.021905 | down | 1.62E-04 | 14.469516 | down |
| TRAIL R1 / DR4 / TNFRSF10A | 6.28E-04 | 17.934732 | up | 3.03E-05 | 30.423908 | up |
| TRAIL R2 / DR5 / TNFRSF10B | 8.90E-04 | 20.22059 | up | 3.22E-07 | 49.28569 | up |
| TWEAK / TNFSF12 | 4.90E-04 | 6.8559923 | up | 0.010095491 | 5.6062865 | up |
| VCAM-1 (CD106) | 0.00159997 | 3.5389478 | down | 0.010093411 | 3.5665014 | down |
| VE-Cadherin | 2.77E-04 | 5.6635346 | up | 5.97E-04 | 5.791487 | up |
| VEGF-B | 0.001706188 | 6.028214 | up | 0.016002305 | 5.36541 | up |
| WIF-1 | 0.033348277 | 2.0870893 | up | 0.046085462 | 2.0818172 | up |

*p-values calculated using *t*-Test. ^†^Regulation: “up” indicates higher levels in stage I or stage III HCC and “down” indicates lower levels in AJCC Stage I or Stage III HCC, compared to NC.
