# Supplementary material for: Identification of Serum Monocyte Chemoattractant Protein-1 and Prolactin as Potential Tumor Markers in Hepatocellular Carcinoma
Source: PLoS One. 2013 Jul 18;8(7):e68904. doi: 10.1371/journal.pone.0068904 (PMC3715515; doi:10.1371/journal.pone.0068904)
Supplement: Table S2 — Association of serum MCP-1 and prolactin levels with patient demographics and disease characteristics in the HCC patients of the SGH cohort (n = 126). (DOC) [file pone.0068904.s003.doc]

**Table S2.** Association of serum MCP-1 and prolactin levels with patient demographics and disease characteristics in the HCC patients of the SGH cohort (n = 126).

| Factor | Median (IQR) | |
| --- | --- | --- |
|  | MCP-1 (ng/ml; IQR^‡^) | Prolactin (ng/ml; IQR^‡^) |
| Gender |  |  |
| Female | 0.65 (0.33, 1.14) | 79.20 (57.32, 101.00) |
| Male | 0.66 (0.38, 1.13) | 65.25 (42.50, 97.80) |
| *p | 0.7028 | 0.2941 |
|  |  |  |
| Hepatitis infection |  |  |
| Hep B/C | 0.78 (0.49, 1.27) | 71.23 (54.28, 105.10) |
| Non Hep B/C | 0.67 (0.37, 1.14) | 67.87 (40.80, 95.14) |
| *p | 0.1866 | 0.5835 |
|  |  |  |
| AJCC Stage |  |  |
| I/II | 0.76 (0.41, 1.17) | 65.76 (40.37, 96.06) |
| III/IV | 1.24 (0.97, 1.79) | 56.12 (40.44, 79.85) |
| *p | 0.0005 | 0.2869 |
|  |  |  |
| Tumour Size |  |  |
| Size ≤ 3cm | 0.58 (0.34, 0.89) | 69.88 (33.74, 100.20) |
| 3cm < Size <10cm | 0.62 (0.37, 1.12) | 77.85 (49.23, 97.89) |
| Size ≥ 10cm | 0.83 (0.49, 1.68) | 57.38 (41.51, 83.22) |
| *p | 0.1183 | 0.4075 |
|  |  |  |
| Cirrhosis |  |  |
| No | 0.61 (0.38, 1.05) | 70.88 (46.39, 99.01) |
| Yes | 0.75 (0.37, 1.18) | 65.07 (41.22, 96.10) |
| *p | 0.9960 | 0.5211 |
|  |  |  |
| PV Thrombosis |  |  |
| No | 0.66 (0.37, 1.17) | 67.18 (42.92, 102.70) |
| Yes | 0.80 (0.53, 0.90) | 74.87 (58.92, 78.50) |
| *p | 0.9784 | 0.7331 |
|  |  |  |
| BCLC scores |  |  |
| A | 0.50 (0.36, 1.00) | 65.90 (43.21, 96.06) |
| B | 0.49 (0.27, 0.65) | 95.43 (56.60, 128.40) |
| C/D | 1.10 (0.95, 1.49) | 60.91 (39.94, 85.22) |
| *p | <0.0001 | 0.2145 |
|  |  |  |
| ALT |  |  |
| ≤ 72 U/L | 0.61 (0.35, 1.12) | 69.88 (42.99, 101.90) |
| > 72 U/L | 0.84 (0.52, 1.24) | 65.25 (42.07, 82.60) |
| *p | 0.1430 | 0.5497 |
|  |  |  |
| AST |  |  |
| ≤ 66 U/L | 0.61 (0.36, 1.07) | 71.90 (44.33, 102.90) |
| > 66 U/L | 0.77 (0.44, 1.33) | 64.78 (40.26, 81.22) |
| *p | 0.1847 | 0.1677 |
|  |  |  |

*p-values calculated using the Mann-Whitney *U* test. ^‡^Interquartile Range (IQR) spans between the 25^th^ percentile and 75^th^ percentile (shown within brackets).
