# Supplementary material for: Identification of Serum Monocyte Chemoattractant Protein-1 and Prolactin as Potential Tumor Markers in Hepatocellular Carcinoma
Source: PLoS One. 2013 Jul 18;8(7):e68904. doi: 10.1371/journal.pone.0068904 (PMC3715515; doi:10.1371/journal.pone.0068904)
Supplement: Table S3 — Summary of univariable analysis for association of factors with recurrence-free survival (RFS). (DOC) [file pone.0068904.s004.doc]

Table S3. Summary of univariable analysis for association of factors with recurrence-free survival (RFS).

| Factor | n | No of events | Median RFS (months) | HR (95% CI) | p-value |
| --- | --- | --- | --- | --- | --- |
| All | 126 | 62 | 29.3 |  |  |
| Age | 126 | 62 | 29.3 | 1.01 (0.99, 1.03) | 0.359 |
| AFP | 120 | 60 | 30.2 | 1.00 (1.00, 1.00) | 0.118 |
| Albumin | 126 | 62 | 29.3 | 0.98 (0.93, 1.03) | 0.430 |
| Bilirubin | 126 | 62 | 30.2 | 1.01 (0.98, 1.04) | 0.600 |
| ALP | 126 | 62 | 29.3 | 1.00 (1.00, 1.01) | 0.028 |
| ALT | 126 | 62 | 29.3 | 1.00 (0.99, 1.01) | 0.832 |
| AST | 126 | 62 | 29.3 | 1.00 (1.00, 1.01) | 0.103 |
| PT | 126 | 62 | 29.3 | 1.25 (1.03, 1.51) | 0.029 |
| Hist Size (cm) | 126 | 62 | 29.3 | 1.00 (0.93, 1.06) | 0.913 |
| MCP-1 (ng/ml) | 125 | 62 | 29.3 | 0.96 (0.64, 1.43) | 0.827 |
| Prolactin (ng/ml) | 125 | 62 | 29.3 | 1.00 (1.00, 1.00) | 0.949 |
| MCP-1 (ULN = 0.62 ng/ml) |  |  |  |  | 0.7318 |
| ≤ULN | 60 | 25 | 22.9 | Reference |  |
| > ULN | 65 | 37 | 33.8 | 0.91 (0.55, 1.53) |  |
| Prolactin (ULN = 83.63 ng/ml) |  |  |  |  | 0.6290 |
| ≤ ULN | 77 | 40 | 30.8 | Reference |  |
| > ULN | 48 | 22 | 22.9 | 1.14 (0.67, 1.93) |  |
| Gender |  |  |  |  | 0.648 |
| Female | 30 | 13 | 47.4 | Reference |  |
| Male | 96 | 49 | 25.4 | 1.15 (0.63, 2.13) |  |
| Race |  |  |  |  | 0.777 |
| Chinese | 99 | 54 | 30.2 | Reference |  |
| Malay | 4 | 1 | 6.7 | 0.59 (0.08, 4.30) |  |
| Others | 23 | 7 | 13.9 | 1.20 (0.54, 2.66 ) |  |
| Hep B |  |  |  |  | 0.971 |
| No | 48 | 24 | 29.3 | Reference |  |
| Yes | 68 | 37 | 25.4 | 1.01 (0.60, 1.69) |  |
| Hep C |  |  |  |  | 0.415 |
| No | 87 | 48 | 30.2 | Reference |  |
| Yes | 10 | 5 | 22.1 | 1.47 (0.58, 3.70) |  |
| AJCC Stages |  |  |  |  | 0.111 |
| 1 | 70 | 35 | 37.5 | Reference |  |
| 3A | 18 | 12 | 9.9 | 1.90 (0.99, 3.68) |  |
| 3B | 1 | 1 | 15.2 | 2.53 (0.34, 18.74) |  |
| Vascular Invasion |  |  |  |  | 0.291 |
| No | 42 | 12 | NR | Reference |  |
| Yes | 26 | 11 | 22.8 | 1.56 (0.68, 3.55) |  |
| Major Branch PV |  |  |  |  | 0.317 |
| No | 118 | 58 | 30.2 | Reference |  |
| Yes | 7 | 3 | 11.8 | 0.56 (0.17, 1.79) |  |
| Cirrhosis |  |  |  |  | 0.282 |
| No | 70 | 30 | 46.1 | Reference |  |
| Yes | 56 | 32 | 25.4 | 1.32 (0.80, 2.17) |  |
| Childs Pugh |  |  |  |  | 0.137 |
| A | 124 | 61 | 30.2 | Reference |  |
| B | 2 | 1 | 5.5 | 4.10 (0.55, 30.72) |  |

*p-values calculated using the Mann-Whitney *U* test. NR, not reached.
