# Supplementary material for: Identification of Serum Monocyte Chemoattractant Protein-1 and Prolactin as Potential Tumor Markers in Hepatocellular Carcinoma
Source: PLoS One. 2013 Jul 18;8(7):e68904. doi: 10.1371/journal.pone.0068904 (PMC3715515; doi:10.1371/journal.pone.0068904)
Supplement: Table S4 — Summary of univariable analysis for association with overall survival (OS). (DOC) [file pone.0068904.s005.doc]

| Factor | n | No of events | Median OS (months) | HR (95% CI) | p-value* |
| --- | --- | --- | --- | --- | --- |
| All | 126 | 44 | 65.0 |  |  |
| Age | 126 | 44 | 65.0 | 1.02 (0.99, 1.05) | 0.146 |
| AFP | 120 | 42 | 66.5 | 1.00 (1.00, 1.00) | 0.126 |
| Albumin | 126 | 44 | 65.0 | 0.97 (0.91, 1.03) | 0.317 |
| Bilirubin | 126 | 44 | 65.0 | 1.01 (0.97, 1.05) | 0.649 |
| ALP | 126 | 44 | 65.0 | 1.01 (1.00, 1.01) | 0.001 |
| ALT | 126 | 44 | 65.0 | 1.00 (0.99, 1.01) | 0.976 |
| AST | 126 | 44 | 65.0 | 1.01 (1.00, 1.01) | 0.085 |
| PT | 126 | 44 | 65.0 | 1.33 (1.07, 1.66) | 0.012 |
| Hist Size (cm) | 126 | 44 | 65.0 | 1.02 (0.95, 1.10) | 0.579 |
| MCP-1 (ng/ml) | 125 | 44 | 65.0 | 0.84 (0.50, 1.39) | 0.490 |
| Prolactin (ng/ml) | 125 | 44 | 65.0 | 1.00 (0.99, 1.01) | 0.986 |
| MCP-1 (ULN = 0.62 ng/ml) |  |  |  |  | 0.3348 |
| ≤ULN | 60 | 19 | 59.5 | Reference |  |
| > ULN | 65 | 25 | 66.5 | 0.74 (0.40, 1.36) |  |
| Prolactin (ULN = 83.63 ng/ml) |  |  |  |  | 0.2336 |
| ≤ ULN | 77 | 26 | 66.5 | Reference |  |
| > ULN | 48 | 18 | 58.0 | 1.45 (0.78, 2.67) |  |
| Gender |  |  |  |  | 0.224 |
| Female | 30 | 7 | 101.2 | Reference |  |
| Male | 96 | 37 | 59.5 | 1.70 (0.72, 4.03) |  |
| Race |  |  |  |  | 0.474 |
| Chinese | 99 | 41 | 59.5 | Reference |  |
| Malay | 4 | 1 | NR | 0.73 (0.10, 5.37) |  |
| Others | 23 | 2 | NR | 0.43 (0.10, 1.79) |  |
| Hep B |  |  |  |  | 0.258 |
| No | 48 | 13 | 66.5 | Reference |  |
| Yes | 68 | 31 | 58.0 | 1.45 (0.76, 2.79) |  |
| Hep C |  |  |  |  | 0.853 |
| No | 87 | 36 | 65.0 | Reference |  |
| Yes | 10 | 2 | NA | 0.87 (0.21, 3.65) |  |
| AJCC Stages |  |  |  |  | 0.003 |
| 1 | 70 | 21 | 76.0 | Reference |  |
| 3A | 18 | 11 | 21.2 | 3.17 (1.51, 6.67) |  |
| 3B | 1 | 1 | 30.5 | 4.63 (0.61, 35.27) |  |
| Vascular Invasion |  |  |  |  | 0.817 |
| No | 42 | 11 | 101.2 | Reference |  |
| Yes | 26 | 7 | 59.5 | 1.12 (0.42, 2.97) |  |
| Major Branch PV |  |  |  |  | 0.131 |
| No | 118 | 40 | 66.5 | Reference |  |
| Yes | 7 | 3 | 57.6 | 2.42 (0.13, 1.35) |  |
| Cirrhosis |  |  |  |  | 0.441 |
| No | 70 | 20 | 59.5 | Reference |  |
| Yes | 56 | 24 | 66.5 | 1.27 (0.69, 2.33) |  |
| Childs Pugh |  |  |  |  | <0.001 |
| A | 124 | 42 | 65.0 | Reference |  |
| B | 2 | 2 | 6.1 | 17.88 (3.76, 85.0) |  |

Table S4. Summary of univariable analysis for association with overall survival (OS).

*p-values calculated using the Mann-Whitney *U* test. NR, not reached; NA, not available.
