# Supplementary material for: Identification of Serum Monocyte Chemoattractant Protein-1 and Prolactin as Potential Tumor Markers in Hepatocellular Carcinoma
Source: PLoS One. 2013 Jul 18;8(7):e68904. doi: 10.1371/journal.pone.0068904 (PMC3715515; doi:10.1371/journal.pone.0068904)
Supplement: Table S5 — Univariable logistic regression analysis results for diagnostic ability of markers. (DOC) [file pone.0068904.s006.doc]

**Table S5.** Univariable logistic regression analysis results for diagnostic ability of markers.

| Factor | Non-HCC HBV carriers (n) | HCC patients (n) | OR (95% CI) | p-value | AUC |
| --- | --- | --- | --- | --- | --- |
| MCP-1 | 115 | 126 | 79.67 (19.56-324.45) | <0.001 | 0.801 |
| Prolactin | 115 | 126 | 1.04 (1.03-1.05) | <0.001 | 0.825 |
| AFP | 110 | 120 | 645.94 (85.28-4892.57) | <0.001 | 0.942 |
